# Not the Stereotypical Wilson Disease: A Case Report

**DOI:** 10.5334/tohm.658

**Published:** 2021-10-29

**Authors:** Amlan Kusum Datta, Adreesh Mukherjee, Jasodhara Chaudhuri, Alak Pandit, Goutam Gangopadhyay

**Affiliations:** 1 Bangur Institute of Neurosciences, Institute of Post Graduate Medical Education and Research, Kolkata

**Keywords:** Wilson disease, Stereotypies, Hyperkinetic movements, MRI Brain

## Abstract

**Background:**

Wilson disease (WD), a potentially treatable genetic disorder with perturbations in copper metabolism, presents with hepatic and neuropsychiatric manifestations. Both hyper and hypokinetic movements predominate the latter spectrum. Motor stereotypies, however, are exceedingly rare.

**Case Report:**

We present a case of a 12-year-old girl, with progressive behavioural alterations and cognitive impairment, with motor stereotypies involving the upper limbs, as the dominant movement semiology. She was diagnosed as WD with evidence of striatal involvement on brain imaging. Her motor symptoms partially responded to chelation therapy.

**Discussion:**

There are about five documented cases of motor stereotypies in WD worldwide, with only one being previously reported from India.

## Introduction

The versatility of the English language, although underscoring its beauty, might be a source of perplexity for the movement disorder specialist. This is best exemplified by the term “stereotypy”, which, in the world of neurology and movement disorders, has been a source of confusion and debate for decades. A unified definition put forth by Edwards et al. [1], considers stereotypies as a purposeless or non-goal directed movement pattern, which occurs in continuum as an invariant loop over a period of time and which is typically distractable. The definition encompasses both intermittent looped motor patterns such as hand flapping/tapping and more severe, repetitive movements which occur at expense of others. Despite obvious phenomenological links with tics, the presence of premonitory urge and the discrete, rapid, and intermittent nature of the motor movement, is believed to distinguish the latter from stereotypies, though this distinction may not always be easy to delineate owing to difficulties in eliciting history of urge from children [1]. Classically, stereotypies have been reported in children with autism, intellectual handicap, sensory deprivation as well as a part of normal developmental process [2]. Although stereotypical gestures are known to be a feature of Wilson disease (WD) as evidenced by its inclusion as an item in the Unified Wilson’s disease rating scale (UWDRS) [3], it is not very common, as previously there has been only 5 reported cases, with only one being reported from India.

## Case description

A 12-year-old girl, born of non-consanguineous marriage, with uneventful birth and developmental history, presented with progressive intellectual decline and difficulty manipulating objects with her hands for past 2 years. She was the only child of her parents and there was no history of neurological illness in her family. There was no history of seizures, loss of consciousness, autonomic dysfunction. She was treatment naïve and there was no history of toxin or drug exposure.

Her facial examination revealed presence of dystonia of forehead involving the corrugator and frontalis muscles. There was also oromandibular dystonia giving rise to a “vacuous smile”. There was evidence of dystonic posturing of hands and toes, which was accentuated on voluntary activity. There were no pyramidal, or cerebellar features nor signs of sensory impairment. Of note, parkinsonian features were absent. Cranial nerve examination revealed slow saccades with intact pursuit, and a dysarthric speech. The most striking feature was the presence of a stereotyped, repetitive, involuntary hyperkinetic movement involving both upper limbs which comprised of flexion, extension of fingers at the metacarpo-phalangeal and interphalangeal joints as well as at wrist, giving rise to a flapping appearance (***Video 1***). It was not associated with urge, nor could the patient suppress it completely on her own volition. It disappeared during sleep and reduced on distraction, without any feeling of distress. It was not associated with any phonic or vocal phenomenon. The patient did not complain of any unpleasant sensation, paraesthesia, or pain of her upper limbs. There was no evidence of motor impersistence as assessed by sustained hand grip, tongue protrusion and eye closure. No frontal release signs were evident. In addition, there was dystonia in the neck and lower limbs, along with chorea in her toes.

**Video 1:** **Stereotypies at presentation**. Demonstration of a repetitive, involuntary, patterned, stereotypical, non-rhythmic movement of both upper limbs involving flexion extension “tapping” movement of fingers as well as “flapping” of both wrists. In addition, a non-rhythmic, side to side “no-no” head movement is noted, occurring independent of the hand movement. As it was suppressible, and not associated with any premonitory urge, it is, in all likelihood, a part of the stereotypical movement. Dystonia of forehead and lower jaw is evident (“vacuous smile”). Dystonic posturing of the toes of feet is also noted along with subtle choreiform movements (especially of left side). Subtle dystonic posturing of fingers of outstretched hands can be appreciated which is otherwise overshadowed by the dominant stereotypical movement.

**Figure 1:**
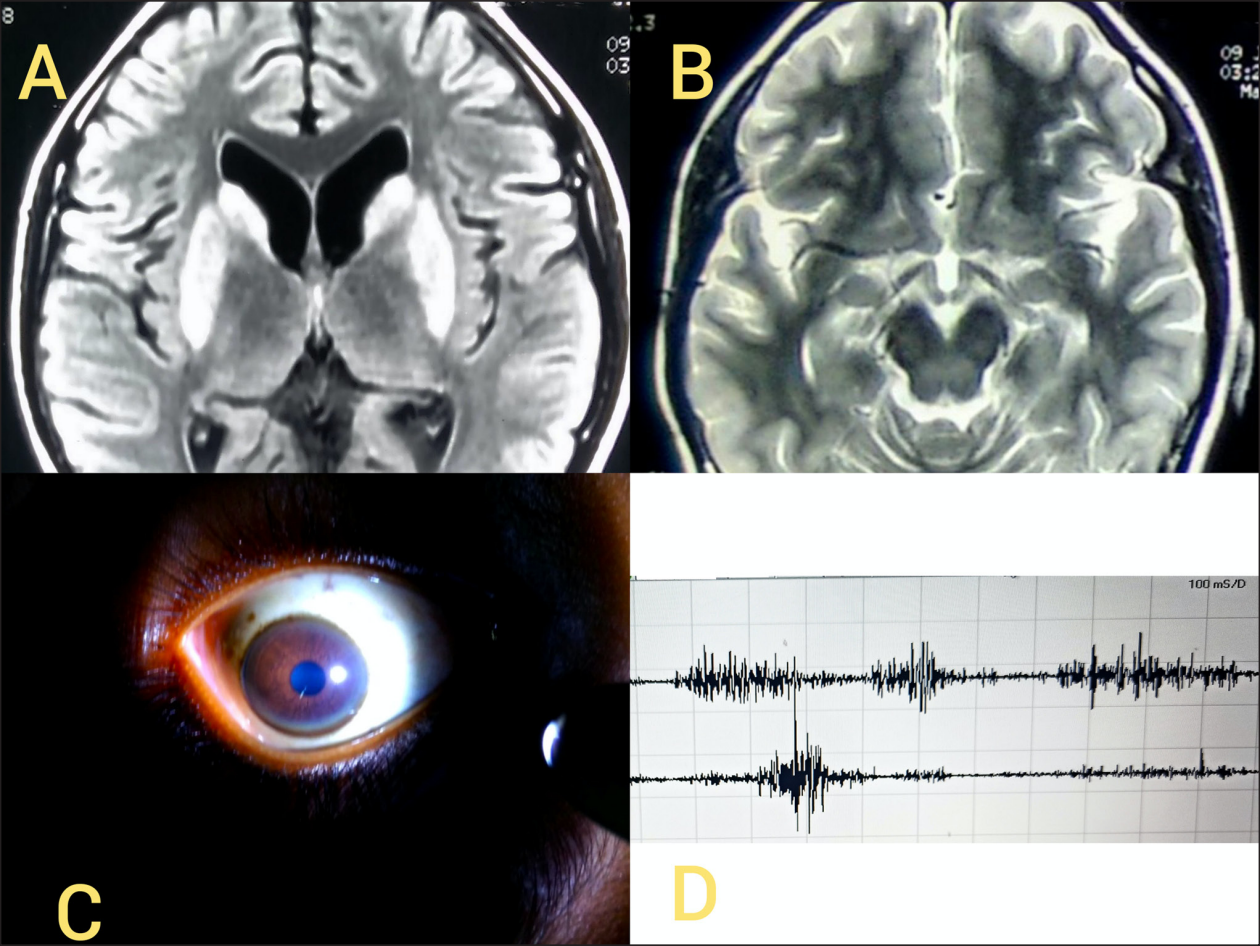
**(A)** MRI Axial T2 weighted fluid attenuation inversion recovery (FLAIR) sequence showing symmetric hyperintensity of bilateral caudate and putamen as well as **(B)** hyperintensities of midbrain. **(C)** Demonstration of brown coloured KF ring in the patient **(D)** Surface EMG of right upper limb of patient with electrodes over Extensor digitorum communis (EDC) and flexor digitorum superficialis (FDS) showing alternating bursts of motor unit action potentials (MUAPs).

Progressive cognitive decline along with hyperkinetic movement semiologies including dystonia, prompted consideration of secondary and heredodegenerative causes, of which WD was at the forefront. Routine investigations, including complete blood counts, hepatic, renal and thyroid function tests were unremarkable. Serum iron and ferritin were within normal limits. Serum ceruloplasmin was low (8 mg/dL; normal range: 20–45 mg/dL) with elevated urinary copper excretion over 24 hours (more than 200 microgram/24 hours). Ophthalmological examination revealed presence of corneal Keyser-Fleischer ring (***Figure 1***). Magnetic resonance imaging (MRI) of brain revealed presence of bilateral, symmetric, putaminal and caudate hyperintensities on T2 weighted sequences (***Figure 1***). Ultrasonography of liver was normal and there were no signs of hepatic dysfunction. Surface electromyography (EMG) of both upper limbs showed alternate bursts of muscle activity over flexor digitorum superficialis and extensor digitorum communis (***Figure 1***). A diagnosis of Wilson disease was made, and patient was started on chelation therapy with penicillamine. Elemental zinc tablets of 50 mg twice daily were also started. Symptomatic therapy was administered with oral clonazepam, trihexyphenidyl and baclofen. At time of writing this manuscript the patient had been followed up for six months. The hyperkinetic stereotyped limb movements had reduced. She was able to manipulate objects and hold pen with her hands, which she was unable to do six months prior. The presence of dystonia of hands as well as neck were better appreciated following reduction of upper limb stereotypies (***Video 2***).

**Video 2:** **Improvement on follow-up**. Follow up video demonstrating reduction of stereotypies. It also shows the presence of dystonia of hands and neck and subtle choreiform toe movements.

## Discussion

WD is an autosomal recessive disease, involving mutations of the ATP7B gene on chromosome 13 [4]. This gene encodes adenosine triphosphatase (ATPase) protein involved in copper transport, perturbations of which leads to excess copper deposition in brain and liver [5].

Movement disorders dominate the neurological spectrum of WD, as noted in a Brazilian series of 119 patients [6], where dystonia, tremor and parkinsonism were the predominant semiologies. A single patient in the series was reported to have a stereotypical movement. A large longitudinal study from India involving 282 patients did not document stereotypies in any patient [7]. The first report of motor stereotypies in a patient of WD was by Yorio AA et al. [8], in 1997. The authors reported on a 26-year-old woman with cirrhosis of liver, presenting with lower limb stereotypies involving toes. The second report was of a 45-year-old Japanese man having purposeless, stereotyped movements of hands [9]. Recently, Bhatti A et al. [10], reported on a 31-year-old male patient with “finger-flapping” hand stereotypy reminiscent of our patient (***Table 1***).

**Table 1:** Illustration of previously reported cases of Wilson disease presenting with stereotypical movements and comparison with present case.


	YORIO AA ET AL., 1997 [7]	OKA Y ET AL., 2002 [8]	BHATTI A ET AL., 2018 [9]	PRESENT CASE

Age/Sex	26Y/Female	45Y/Male	31Y/Male	12Y/Female

Stereotypical movement	Involuntary, patterned, repetitive movement of feet	Stereotypical, purposeless hand movements such as rubbing and clapping behind back	Finger flapping	Finger flapping

Brain imaging	Bilateral caudate, putamen, thalamic and upper brainstem T2 hyperintensity	Bilateral pallidal and brainstem involvement	Brain MRI showed abnormal T2 hyperintensities in the brainstem, with normal basal ganglia. FDG PET scan revealed severe hypometabolism in the basal ganglia	T2 hyperintensities in basal ganglia and midbrain


The movement described in the present case, was non-rhythmic, and of low frequency, ameliorated on distraction, unlike tremor, which is typically rhythmic and usually does not improve on distraction. The patterned, seemingly purposeless, repetitive nature of the movement was distinct from choreiform movements which are, by definition, random as well as quasi-purposive. Albeit absence of premonitory urge or sensation is theoretically, a hallmark differentiating feature between tics and other hyperkinetic motor semiologies including stereotypies, the history is often difficult to discern, particularly in the paediatric age group. In the present case, the fixed, almost identical pattern of movement as well as the prolonged duration distinguished it from tics, which are more variable, and short-lasting. Suppressibility following external distraction without being associated with any discomfort favoured stereotypy over motor tics.

Neurobiological studies in lower animals as well as functional scans have implicated the cortico-striatal-thalamo-cortical (CSTC) circuit in the basic pathophysiology of repetitive, stereotypical, motor movements. Reduced levels of extracellular dopamine and increased acetylcholine in the vicinities of prefrontal region of dorsal striatum as well as anterior cingulate gyrus are consistent findings in individuals with motor stereotypies [11]. In light of this concept, a theoretical possibility remained, that administration of trihexyphenidyl for control of dystonia might also help ameliorate the stereotypical movements. However, taking in account the time lag for improvement of the latter, the therapeutic response perhaps would be better attributed to chelation rather than anti-cholinergic. Presumably, the pathophysiological substrate for stereotypy may involve far more intricate neurobiochemical shifts involving numerous neurotransmitters, that may be beyond our present understanding.

In conclusion, it is well accepted that WD is a great masquerader, with a vast spectrum of neurological and non-neurological symptomatologies. Albeit rarely described in WD, stereotypies are common in childhood and recognition of its key features are essential to differentiate them from other hyperkinetic semiologies as well as to avoid missing a potentially treatable underlying cause.
